# Micro-CT image gallery visually presenting the effects of ocean warming and acidification on marine gastropod shells

**DOI:** 10.3897/BDJ.9.e75358

**Published:** 2021-12-07

**Authors:** Eva Chatzinikolaou, Kleoniki Keklikoglou, Panagiotis Grigoriou, Christos Arvanitidis

**Affiliations:** 1 Hellenic Centre for Marine Research (HCMR), Institute of Marine Biology, Biotechnology and Aquaculture (IMBBC), Heraklion, Crete, Greece Hellenic Centre for Marine Research (HCMR), Institute of Marine Biology, Biotechnology and Aquaculture (IMBBC) Heraklion, Crete Greece; 2 Biology Department, University of Crete, Heraklion,Crete, Greece Biology Department, University of Crete Heraklion,Crete Greece; 3 Cretaquarium, Hellenic Centre for Marine Research, Heraklion, Crete, Greece Cretaquarium, Hellenic Centre for Marine Research Heraklion, Crete Greece; 4 LifeWatch ERIC, Seville, Spain LifeWatch ERIC Seville Spain

**Keywords:** micro-CT, 3D image galleries, gastropod, shell, *Hexaplextrunculus*, climate change, ocean acidification, ocean warming

## Abstract

**Background:**

Digitisation of specimens (e.g. zoological, botanical) can provide access to advanced morphological and anatomical information and promote new research opportunities. The micro-CT technology may support the development of "virtual museums" or "virtual laboratories" where digital 3D imaging data are shared widely and freely. There is currently a lack of universal standards concerning the publication and curation of micro-CT datasets.

**New information:**

The aim of the current project was to create a virtual gallery with micro-CT scans of individuals of the marine gastropod *Hexaplextrunculus*, which were maintained under a combination of increased temperature and low pH conditions, thus simulating future climate change scenarios. The 3D volume-rendering models created were used to visualise the structure properties of the gastropods shells. Finally, the 3D analysis performed on the micro-CT scans was used to investigate potential changes in the shell properties of the gastropods. The derived micro-CT 3D images were annotated with detailed metadata and can be interactively displayed and manipulated using online tools through the micro-CT virtual laboratory, which was developed under the LifeWatchGreece Research Infrastructure for the dissemination of virtual image galleries collection supporting the principles of FAIR data.

## Introduction

The need for access to accurate virtual representations of species has been mentioned by several authors ([Bibr B7446188][Bibr B7446179]). Museums and academic institutions increasingly recognise the value of specimen digitisation as a means to boost the impact of collections for research and society through limitless information access and use (Beaman and Cellinese 2012). Virtual representations of species or type materials, also called "cybertypes", can provide new research opportunities through the access to anatomical and morphological information (Faulwetter et al. 2013). Micro-computed tomography (micro-CT) may support the development of "virtual museums" or "virtual laboratories" as an imaging technology, where digital data (and images as data) are widely and freely shared (Abel et al. 2011, Keklikoglou et al. 2016). Micro-CT is a non-destructive imaging technique which allows the creation of 3D morphological data and allows the full virtual representation of both internal and external features of the scanned specimen at micrometre resolution (Faulwetter et al. 2013). Micro-CT has the ability to create 3D virtual representations of samples which can contribute to the aforementioned needs. There are currently no standards concerning the publication of micro-CT datasets, since their size is large and there are no rules and guidelines for data curation and documentation of such datasets (Keklikoglou et al. 2016, Keklikoglou et al. 2019). Additionally, no universal standards of micro-CT metadata exist, while the DICOM protocol is mainly applicable to medical sciences (Digital Imaging and Communications in Medicine; http://dicom.nema.org).

## General description

### Purpose

The aim of the project was to investigate the effect of ocean warming and acidification, as a result of climate change, on the shell structure of the marine gastropod *Hexaplextrunculus*, using a non-destructive 3D imaging technique. Furthermore, this data paper presents an example of how the micro-CT datasets can be shared and retrieved in order to contribute to the massive digitisation and open access of biological collections.

The higher levels of greenhouse gas emissions are mainly responsible for the increase observed in the global average sea surface temperature during the last 60 years (IPCC 2014). More specifically, the upper 75 m of the sea surface has been experiencing a warming of 0.11ºC per decade over the period 1971 to 2010 (IPCC 2014). At the same time, oceans have absorbed approximately 25% of the CO_2_ released into the atmosphere by humans since the start of the Industrial Revolution, which has resulted in a reduction of the ocean pH by 0.1 units (IPCC 2014). Climate change and ocean acidification are affecting the shell structure of calcifying marine organisms, such as gastropods because the chemistry of the ocean is altered, thereby leading to a reduction in the calcium carbonate saturation state (Kleypas et al. 2006).

Individuals with calcified structures are able to adjust their shell properties under ocean acidification conditions, mainly the ones related to thickness and packing (i.e. porosity) of calcium carbonate crystals, in order to build more resilient shells (Leung et al. 2020). Shell features, such as density, thickness and porosity, can thoroughly describe alterations in shell integrity and compaction and, therefore, indicate the ability of gastropods to successfully adapt to future climate change challenges. Visual comparisons between different micro-CT scans revealed that the shell of individuals maintained under acidified conditions (low pH) had more transparent (i.e. less dense) areas and an increased number of closed pores.

The micro-CT image datasets of the present study were stored in the Micro-CT virtual laboratory (micro-CT vLab), which is developed under the LifeWatchGreece ESFRI Research Infrastructure and supports the integration of imaging data into a large European Infrastructure. The micro-CT vLab hosts and disseminates micro-CT virtual image galleries with 3D specimens of various taxa, annotated with detailed metadata through a catalogue API service. The users are able to either retrieve the datasets or to interact with the 3D models by using a series of online tools giving the opportunity to virtually dissect the samples. All scans have been performed using a Skyscan 1172 microtomograph (Bruker, Kontich, Belgium) at the Hellenic Centre for Marine Research (HCMR). The cross-section images can be used to create 3D volume rendering images and videos of specimens. The wide dissemination of these "cyber-specimens" aims to contribute to a massive digitisation and open access of biological collections, thus contributing to the well recognised FAIR data principles.

## Project description

### Title

Effects of climate change and ocean acidification on marine gastropods (ECCO project)

### Personnel

Dr Eva Chatzinikolaou (scientific responsible, experimental design, sample scanning, image analysis, data management), Kleoniki Keklikoglou (sample scanning, image analysis, data management), Dr Panagiotis Grigoriou (experimental design).

### Study area description

Mediterranean Sea, Greece, Crete

### Design description

Individuals of the gastropod *Hexaplextrunculus* (Linnaeus, 1758) were collected from shallow (< 5 m) soft bottom habitats (Crete, Greece), acclimatised in laboratory conditions for a month and then equally separated into four different experimental treatments (55-60 individuals per treatment; size range 13.2-70.9 mm): a) Control (normal pH = 8.1 and ambient temperature, b) Acid (low pH = 7.8 and ambient temperature, c) Warm (normal pH = 8.1 and increased temperature +3ºC) and d) Warm and acid (low pH = 7.8 and increased temperature +3ºC). The experimental treatments were representing the RCP8.5 "high GHG emission" scenario according to the Intergovernmental Panel for Climate Change (IPCC 2014). The experiment lasted for 12 months

*Hexaplextrunculus* was selected as the model species for this experiment since it is a common and widely distributed sublittoral gastropod, which is well-adjusted to varying physical environmental conditions characterising transitional coastal systems (e.g. rock pools, lagoons) where temperature and pH fluctuations occur naturally (Wahl et al. 2016). *Hexaplextrunculus* has been widely used as a Tributyltin (TBT) pollution bioindicator and is also an edible species with important economic value in several countries (Abidli et al. 2009).

### Funding

This work was funded under the project ECCO (HFRI, Hellenic Foundation for Research and Innovation for the support of Post-doctoral Researchers, project ID 343).

## Sampling methods

### Sampling description

A group of six individuals per treatment were randomly sampled for scanning in the micro-CT after the gastropods were maintained for 12 months under the experimental conditions.

### Quality control

One of the four experimental treatments was the Control (ambient temperature, normal pH) which serves as a quality control measure for the experimental design. Six randomly-selected replicates (specimens) were scanned from each treatment in order to adjust for any possible variability between individual gastropods. A 1:1 ratio of female:male was selected for the scanned specimens.

### Step description

The following steps were followed for scanning and analysis of the selected specimens:

1. Anaesthetisation of *Hexaplextrunculus* gastropods with 7% MgCl_2_ and storage at -20°C.
2. Scanning of samples without any staining using a SkyScan 1172 micro-tomograph (Bruker, Kontich, Belgium) at the Hellenic Centre for Marine Research (HCMR), Institute of Marine Biology, Biotechnology and Aquaculture (IMBBC), Heraklion, Crete, Greece.
3. Reconstruction of projection images into cross sections using the SkyScan’s NRecon software (Bruker, Kontich, Belgium).
4. Creation of volume renderings of each specimen using the CTVox software (Bruker, Kontich, Belgium).
5. Calculation of the mean grey scale values of the total shell using the binary threshold module of the software CT Analyser (CTAn, Bruker, Kontich, Belgium), which allows for comparable measurements of the relative density of the shell. Relative grey scale density was used as a proxy for estimating "micro-density" (i.e. density of the shell material including CaCO_3_ and intraskeletal organic matrix, excluding porosity).
6. 3D analysis using the custom processing plugin of the CTAn software in order to calculate the porosity and the structure thickness for each specimen. Porosity was calculated as the percentage of the closed porosity of the shell (i.e. total volume of enclosed pores of each specimen as a percentage of the total shell volume). Structure thickness of the shell was calculated as the average of the diameters of the largest spheres which can be fitted into each point of the shell structure ("sphere-fitting" method) (Hildebrand and Rüegsegge 1997).

The parameters of the scanning, reconstruction and analysis procedure remain the same amongst the different treatments in order to obtain comparable results.

The detailed protocols for *Hexaplextrunculus* scanning and analysis have been published and received a DOI under https://dx.doi.org/10.17504/protocols.io.bxwqppdw.

## Geographic coverage

### Description

The collection of samples was performed in Elounda, Crete, Greece (N 35° 16' 2.4'', E 25° 43' 27.2'').

## Taxonomic coverage

### Description

Phylum: Mollusca, Class: Gastropoda, Order: Neogastropoda, Family: Muricidae, Genus: *Hexaplex*, Species: *Hexaplextrunculus*

## Traits coverage

Non-applicable.

## Collection data

### Collection name

ECCO project - *Hexaplextrunculus*

### Specimen preservation method

Frozen at -20ºC

### Curatorial unit

Institute of Marine biology, Biotechnology and Aquaculture (IMBBC) - Hellenic Centre for Marine Research (HCMR)

## Usage licence

### Usage licence

Other

### IP rights notes

Creative Commons Attribution 4.0 International License

## Data resources

### Data package title

ECCO project data - *Hexaplextrunculus*

### Resource link

https://microct.portal.lifewatchgreece.eu/

### Number of data sets

1

### Data set 1.

#### Data set name

Effect of increased temperature and low pH on *Hexaplextrunculus*

#### Data format

NIfTI image files (Neuroimaging Informatics Technology Initiative) is a data format for the storage of Functional Magnetic Resonance Imaging (fMRI) and other medical images.

#### Number of columns

32

#### Download URL

https://microct.portal.lifewatchgreece.eu/node/72, https://microct.portal.lifewatchgreece.eu/node/71, https://microct.portal.lifewatchgreece.eu/node/70, https://microct.portal.lifewatchgreece.eu/node/69

#### Description

The dataset is available through the Micro-CT vLab hosted in the Institute of Marine Biology, Biotechnology and Aquaculture (IMBBC) of the Hellenic Centre for Marine Research (HCMR). The current publication aims to describe the 3D image galleries produced during the micro-CT scanning of specimens derived from the four ECCO project experimental treatments: 1) *Hexaplextrunculus* in normal conditions (https://microct.portal.lifewatchgreece.eu/node/70), 2) *Hexaplextrunculus* in acidified conditions (https://microct.portal.lifewatchgreece.eu/node/69), 3) *Hexaplextrunculus* in warm conditions (https://microct.portal.lifewatchgreece.eu/node/71) and 4) *Hexaplextrunculus* in warm and acidified conditions (https://microct.portal.lifewatchgreece.eu/node/72). These four galleries (i.e. "*Hexaplextrunculus* projects") are freely available for downloading and online manipulation through the  LifeWatchGreece web portal interface.

**Figure 1.**
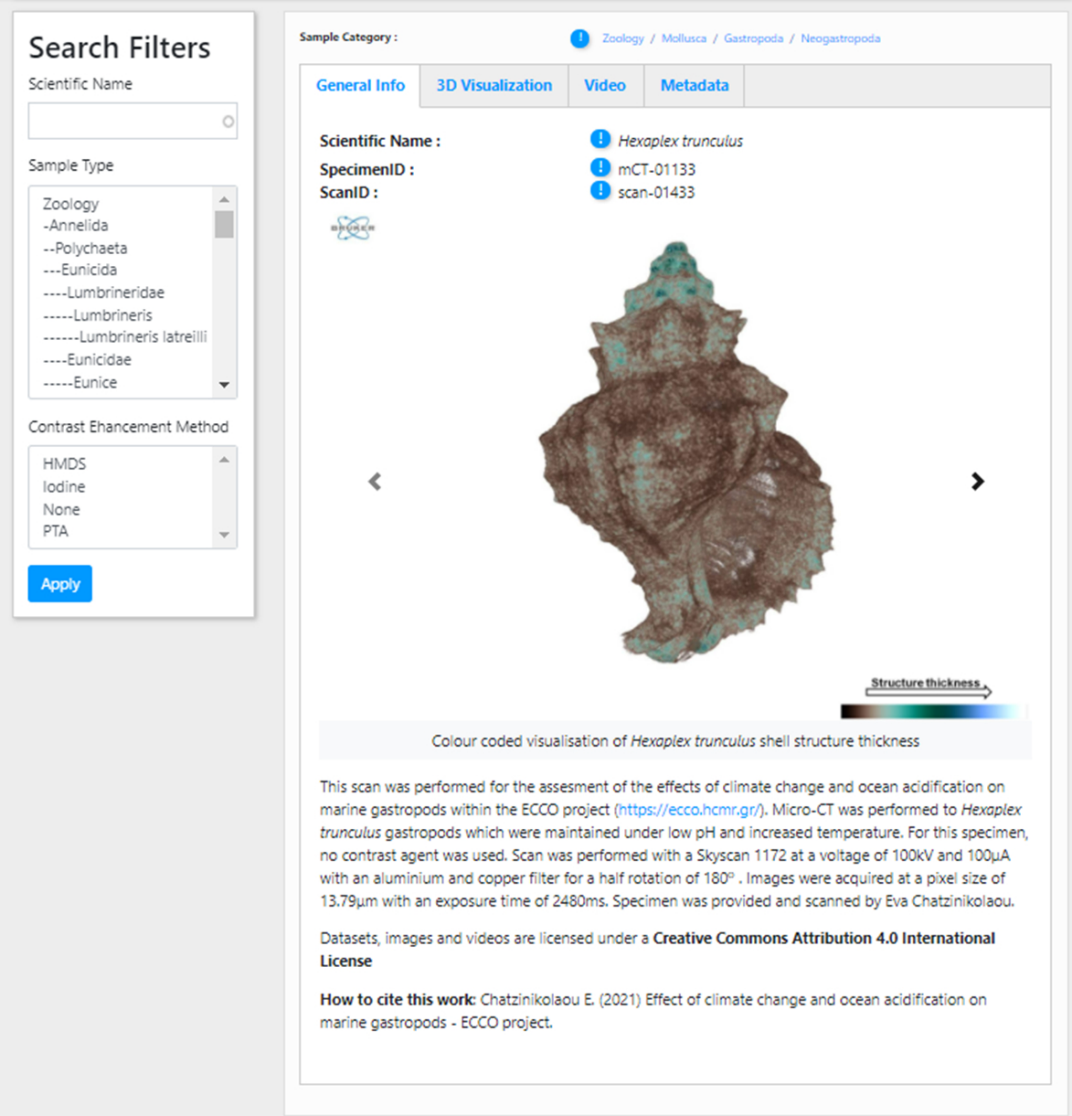
The General Info tab in the Micro-CT vLab.

The micro-CT gallery for each "project" includes a General Info tab (Fig. 1) where the specimen and scan IDs are shown, together with the taxonomic classification of the species. In addition, a short description of the specimen, the experimental treatment (aim of scanning) and the scanning parameters (e.g. contrast agent, voltage, current, filter, rotation, resolution, exposure time, specimen and scan provider) are indicated. Three images are presented for each specimen/treatment in the General Info tab: a) volume rendering of the shell, b) closed porosity of the shell and c) colour-coded visualisation of the shell structure thickness. The type of Creative Common Attribution licence and the appropriate citation applied for the specific dataset can be seen at the bottom of the page.

**Figure 2.**
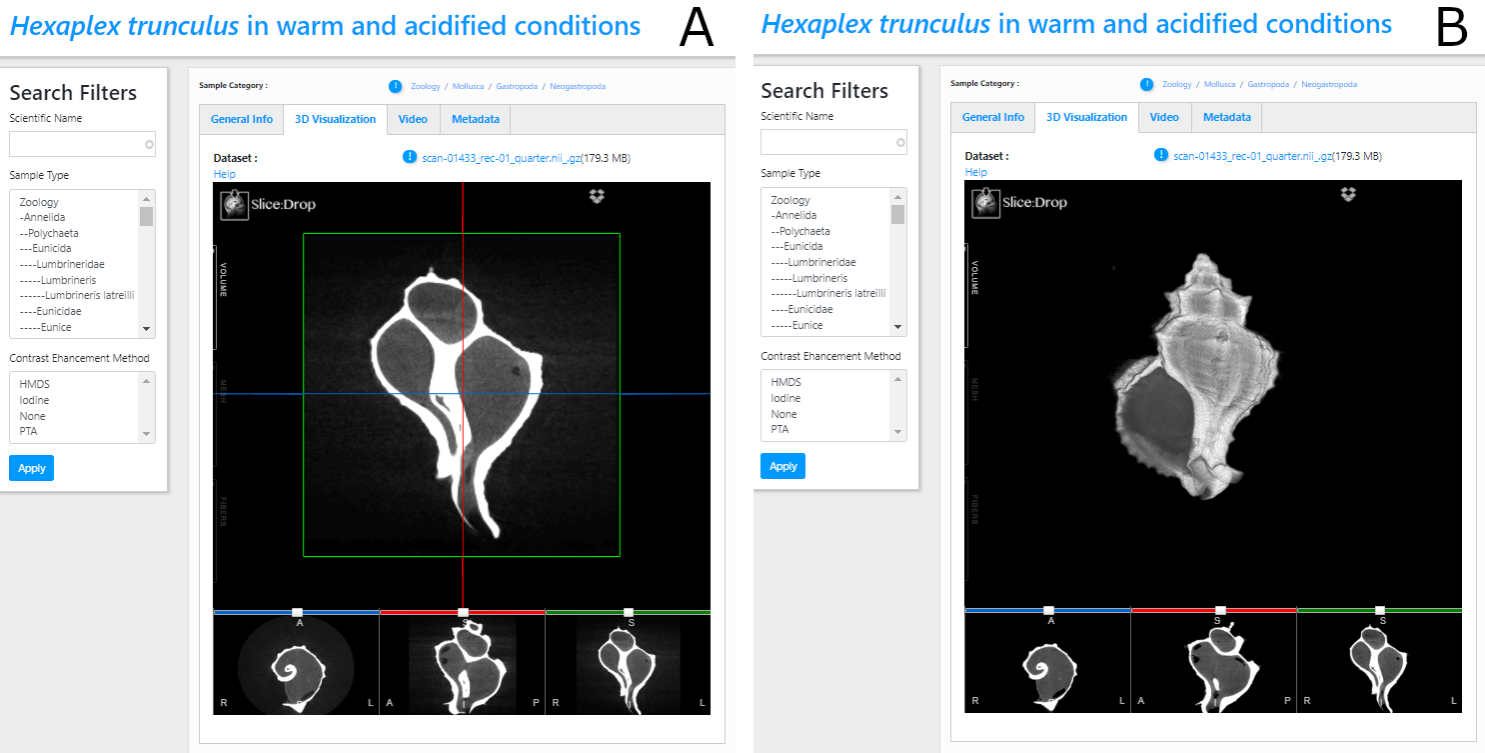
A) micro-CT slices in three orthogonal views (x, y, z axes), B) volume rendering of the micro-CT scan using the Slice:Drop software.

In the 3D visualisation tab, a "volume" slider appears on the upper left side of the panel, which enables the display of the selected micro-CT dataset either as 2D or 3D using the Slice:Drop software. The user can explore the micro-CT dataset in 2D and view all the micro-CT slices in three orthogonal views (x, y, z axes) (Fig. 2A). Selection of the 3D icon in the "volume" slider tab (sliding on the left side of the panel) displays the 3D volume of the specimen (Fig. 2B). The user has the ability: a) to rotate this 3D specimen, b) to change the opacity (transparency parameters), c) to alter the thresholding parameters (i.e. to "compress" the grey-scale values so that only the most dense parts or all parts are visible) and d) to colour the specimen and thus create contrasts with the different colours.

**Figure 3.**
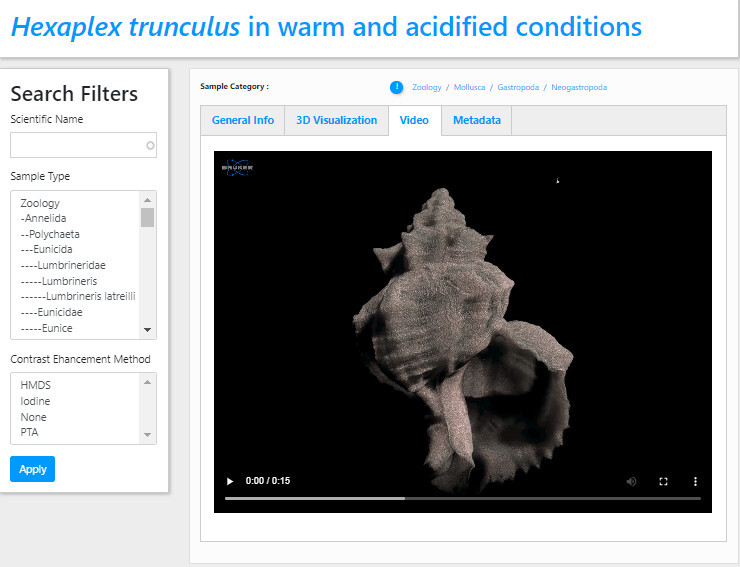
The preview video of a *Hexaplextrunculus* micro-CT scan.

The Video tab displays a short preview video as a demonstration of the specific micro-CT dataset (Fig. 3), featuring the morphology and anatomy of the internal and external features of the selected *Hexaplextrunculus* specimen.

The Metadata tab contains complete detailed information about the dataset, the pre-scanning procedures and the scanning parameters, following the schema proposed by Faulwetter et al. (2015). An info icon is located next to each metadata term offering an explanation for the description of each field (see Table below). At the bottom of the Metadata tab, the user is able to download: a) the full micro-CT dataset (i.e. 3D volume rendering of cross section images in NIfTI format) of the selected specimen/scan (see field "Dataset"), b) the image files (jpg format) for structure thickness and closed porosity of the shell (see field "Micro-CT images") and c) a video file (mp4 format) provided for each specimen (see field "Video file"). The type of Creative Common Attribution licence applied for the specific dataset can be seen at the bottom of the page.

**Table**: Metadata terms of micro-CT vLab datasets and their definitions.

**Data set 1.** 

Column label	Column description
Specimen ID	A unique identifier for the specimen in the format mCT-xxxxx (where x = incrementing number from 00001 to 99999, with preceding zeros).
Scan ID	A unique code of the format scan-xxxxx (where x = incrementing number from 00001 to 99999, with preceding zeros).
Sample Category	The category to which the specimen belongs, for example, Zoology, Botany.
Scientific name	The lowest taxonomic name to which the specimen has been identified.
Taxonomic Group	The general taxonomic group to which the specimen belongs, for example, Polychaeta, Insecta
Specimen Description	A verbatim description of the specimen, which allows the understanding of the nature of the specimen at a glance.
Provider Institute	Institution (i.e. academic, scientific) which provided the specimen.
Specimen Provider	Name of the person who provided the specimen.
Material	The material of the scanned sample, for example, soft tissue.
Fixation Type	Type of chemical used for the fixation of the specimen, for example, formalin.
Preservation Medium	Means used for preservation of the specimen, for example, ethanol, freezer.
Contrast Enhancement Method	Contrast agent (short name of the chemical, for example, PTA) used in order to achieve contrast difference between the specimen and its surrounding medium.
Scope of Scan	Reason for performing a scan.
Scan date	Start date of the scanning in the format MM/DD/YYYY.
Scanned By	The name of the person who performed the scan.
Sample Holder	A description of the sample holder, for example, pipette tip.
Scanning Medium	The medium that surrounds the sample during scanning, for example, air, ethanol.
Scanned Part	The part of the specimen that has been scanned, for example, anterior part, full specimen.
Digital Device Type	The brand (manufacturer) of the Digital Device that was used for the scanning.
Voltage kV	The voltage of scanning in kilovolt (kV).
Current μA	The current of scanning in μAmpere.
Filter	The type of the filter that is used for scanning, for example, Aluminium
Zoom (μm)	The resolution of the scan in μm (zoom level), for example, 1.24.
Camera Resolution	Camera resolution settings in pixels, for example, 4000.
Exposure Time (ms)	Duration of time (milliseconds) the sample is exposed under X-rays.
360	Full (360°) or half (180°) rotation of the specimen during scanning.
Random Movement	The maximum number of pixels to randomly move the specimen up and down in order to avoid "dead pixels".
Averaging	The number of images acquired for each position (angle) in order to estimate the frame averaging value.
Oversize Settings	The number of parts (vertical & horizontal) used for scanning of oversize specimens.
Dataset	Download the dataset in NIfTI format.
Micro-CT Images	Download the micro-CT images.
Video File	Download the micro-CT video in mp4.
